# CC-Chemokine Ligand 18 Induces Epithelial to Mesenchymal Transition in Lung Cancer A549 Cells and Elevates the Invasive Potential

**DOI:** 10.1371/journal.pone.0053068

**Published:** 2013-01-18

**Authors:** Till Ploenes, Ben Scholtes, Alexander Krohn, Meike Burger, Bernward Passlick, Joachim Müller-Quernheim, Gernot Zissel

**Affiliations:** 1 Division of Thoracic Surgery, University, Medical Centre Freiburg, Freiburg, Germany; 2 Division of Internal Medicine, Department of Pneumology, University Medical Centre Freiburg, Freiburg, Germany; 3 Division of Internal Medicine, Department of Oncology and Hematology, University Medical Center Freiburg, Freiburg, Germany; Cincinnati Children's Hospital Medical Center, United States of America

## Abstract

Lung cancer is one of the leading causes of cancer related death worldwide with more than a million deaths per year. The poor prognosis is due to its high aggressiveness and its early metastasis. Although the exact mechanisms are still unknown, the process of epithelial to mesenchymal transition (EMT) seems to be involved in these neoplastic processes. We already demonstrated that serum levels of CCL18, a primate specific chemokine, are highly elevated in patients with lung cancer and correlate with their survival time of patients with adenocarcinoma of the lung. Therefore, we hypothesized that CCL18 may be directly involved in pathological processes of lung cancer, e.g. EMT. We investigated the effect of CCL18 on A549, an adenocarcinoma cell line of the lung, on EMT and other cell functions like proliferation, chemotaxis, invasion, chemoresistance and proliferation. Exposure of A549 lung cancer cells to CCL18 in various concentrations decreases the epithelial marker E-cadherin, whereas FSP-1, a marker of the mesenchymal phenotype increases. Accordingly, CCL18 induced the transcriptional EMT regulator SNAIL1 in a dose dependent fashion. In contrast, an increasing CCL18 concentration was associated with a decline of cell proliferation rate. In addition, CCL18 induced chemotaxis of these cells and increased their chemoresistance. Therefore, CCL18 may be an interesting therapeutic target for NSCLC.

## Introduction

Tumor metastasis is a complex event involving multiple steps including separation of cancer cells from the compact primary tumor, migration into vessels, invasion in tissue and formation of a secondary tumor nodule. Although still under debate, epithelial to mesenchymal transition (EMT) seems to be one of the key events in local progress and metastasis of epithelial malignancies [1], [2]. EMT is a complex multistep event, which changes not only cell morphology but also enables cells to gain important new functions like the expression of new molecules or migration and invasion. In addition to morphological changes, the process of EMT is characterized by differences in transcription and expression of epithelial and mesenchymal genes. One of the most important molecular markers of epithelial cells is the epithelial adhesion molecule E-cadherin, mediating cell-cell interactions [3]. The process of EMT is associated with a loss of E-cadherin, which is positively correlated with tumor stage and poor survival in many epithelial tumor [4]–[6]. Beside the loss of epithelial markers the expression of mesenchymal markers like FSP-1 is also an important step in the process of EMT [7]. FSP-1, also called S100A4, correlates with the metastatic potential in neoplasms and some authors demonstrated that a high level of FSP-1 is associated with poor prognosis in various cancer types [8]–[10]. EMT is regulated by several transcription factors [11]. One of the most important is SNAIL1, which acts also as E-cadherin repressor. It has been shown that high SNAIL1 expression is associated with poor prognosis in lung cancer [12]. EMT can be induced by several growth factors and cytokines, most importantly by TGFbeta but also by EGF, FGF, HGF and others [13]. Most of these factors are present in the tumor environment and produced by the tumor cells itself or by surrounding cellular components. The microenvironment of solid tumors is a complex mixture of cellular and non cellular factors, in which the tumor associated macrophages (TAM) represents up to 50% of the tumor mass [14]. TAMs are alternatively activated macrophages secreting a specific pattern of several tumor promoting cytokines and growth factors. One of these cytokines is the human specific CC-Chemokine Ligand 18 (CCL18) which is highly present in lung tissue and involved in several pathological processes of malignant diseases or fibrosis [15]–[20]. We already demonstrated that CCL18 is highly elevated in sera of patients with non small cell lung cancer and correlates with tumor stage and overall survival in the subgroup of adenocarcinoma [21], [22]. Mean CCL18 serum level of the patients with non-small-cell lung cancer was 150(857) ng/ml vs. 32(61) ng/ml in the healthy control group [22]. We, therefore, hypothesized that CCL18 is an inducer of EMT in human adenocarcinoma cells of the lung and enhances the metastatic potential.

## Materials and Methods

### Reagents

Recombinant human TGFbeta1 was purchased from R&D (R&D Systems, Wiesbaden, FRG), recombinant human CCL18 from Immunotools, Friesoyte, Germany, polyclonal rabbit antibodies against human FSP-1 and human SNAIL1 and a mouse monoclonal antibody against human tubulin were purchased from abcam (abcam, Cambridge, UK) and a polyclonal rabbit antibody against human E-cadherin was from upstate (upstate, Billerica, USA).

### Cell Culture Experiments

Human adenocarcinoma cell line A549 (ATCC, CCL-185) was cultured in Dulbecco's modified Eagles medium (DMEM, Invitrogene, Carlsbad, U.S.A) with 10% fetal bovine Serum (PAA, Pasching, Austria), 100 µg/ml Streptomycin and 100units/ml penicillin (Invitrogen) at 37°C in a humidified 5% CO_2_ atmosphere. Cultures of cells were harvested at 80% confluence 24 hours before stimulation, counted and seeded in six well plates at a density of 30,000 cells per ml. Cells were incubated with CCL18 in various concentrations (100 pg/ml, 1 ng/ml, 10 ng/ml, 100 ng/ml) for 24 hours or 72 hours in serum-free DMEM. TGFbeta (2 ng/ml, ) was used as positive control.

### Reverse Transcription PCR (RT-PCR)

Total RNA was isolated using Trizol (Invitrogen). Reverse transcription was performed using Omniscript RT Kit (Qiagene, Düsseldorf, FRG). Specific primers for human SNAIL1, FSP-1, E-cadherin and GAPDH were designed using AmplifX software (http://ifrjr.nord.univ-mrs.fr/AmplifX-Home-page) using NLM GenBank databases (National Center for Biotechnology Information; http://www.ncbi.nlm.nih.gov/nuccore/). Accession code numbers for the nucleotide sequences used to generate the respective primers and the primer sequences are listed in table 1.

**Table 1:** PCR Primer.

*Target Gen*	*Accession code*	*Primer Sequence forward (5′-3′)*	*Primer Sequence reverse (5′-3′)*
GAPDH	NM_002046	CAC CAG GGC TGC TTT TAA CT	GAT CTC GCT CCT GGA AGA TG
E Cadherin	NM_004360	TGC CCA GAA AAT GAA AAA GG	GTG TAT GTG GCA ATG CGT TC
FSP1	NM_019554	GGT GTC CAC CTT CCA CAA GT	GCT GTC CAA GTT GCT CAT CA
Snail	NM_005985	GCG AGC TGC AGG ACT CTA AT	GGA CAG AGT CCC AGA TGA GC

All primers were intron-spanning and synthesized by Biomers (Biomers.net, Ulm, FRG). Real time PCR was performed using iQ SYBR Green SuperMix, iCycler thermocycler, and iCycler iQ 3.0 software (Bio-Rad Laboratories GmbH, München, FRG) according to the manufacturer's protocol. Melting curve analysis was used to control for specificity of the amplification products. No amplification of nonspecific products was observed in any of the reactions. A threshold cycle value (Ct) was calculated and used to compute the relative level of specific mRNA in our samples by the following formula:
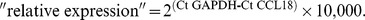



### Western Blot

Cells were washed three times with ice-cold PBS and scraped from the bottom. The cells were lysed with lysis buffer (50 mM Tris-HCl pH 7,6, 150 mM NaCl, 2 mM EDTA, 2 mM EGTA, 0,1% Trition-X) containing a protease inhibitor cocktail. Total protein concentration was measured using BCA protein assay kit (Thermo Scientific, Waltham, MA) with bovine serum albumin as standard. Whole cell lysates were boiled at 93°C for 5 minutes in equal volumes of loading buffer (0,5 M Tris-HCl pH 6.8, 2% SDS, 0, 05% bromphenol-blue, 20% 2-mercaptoethanol, 10% Glycerol).

All samples were subjected to 12% sodium dodecylsulfate–PAGE, separated by electrophoresis and transferred to a polyvinylidene difluoride membrane (PVDF). After blocking for 2 h in Tris-buffered saline (TBS) containing 5% non-fat dry milk, the membranes were incubated with primary antibody diluted 1∶200 (FSP-1), 1∶500 (E-cadherine) or 1∶3000 (SNAIL1) with TBS containing 5% non-fat dry milk at 4°C overnight. Visualization was performed using appropriate secondary antibodies labeled with IRDye 800CW or IRDye 700CW (Li-COR Bioscience, Bad Homburg, FRG) diluted 1∶10 000–1∶20000 for 2 h and scanned using Odyssey system (Li-COR Bioscience, Bad Homburg, FRG) according to the manufactures instructions.

### Chemotaxis Assay

Chemotaxis assays were performed using a 10-well chemotaxis chamber (NeuroProbe, Gaithersburg, U.S.A.). The bottom wells were filled with 400 µl of DMEM containing CCL18 in concentrations of 100 pg/ml, 1 ng/ml, 10 ng/ml, and 100 ng/ml (control: TGFbeta 2 ng/ml). A 12 µm-pore-diameter polyvinylpyrrolidone-free polycarbonate filter (NeuroProbe, Gaithersburg, U.S.A.) was placed on the bottom plate. A silicon gasket and the top plate with 12 holes were then mounted, forming the top wells. 2×10^4^ cells were added in a volume of 285 µl. In parallel experiments, DMEM without chemokine was loaded into the bottom wells as a negative control. A second negative control with chemokine of the same concentration in the upper and the lower well was also performed. After 24 hours of incubation at 37°C and 5% CO2, the filter sheet was removed, and non-migrated cells were wiped off from the top side of the filter. The filter was then stained using crystal violet (Sigma-Aldrich, St. Louis, U.S.A.) and fixed in clear lacquer. After this 9 high-power fields per filter were counted at 200-fold magnification using a Zeiss microscope.

### Invasion Assay

The ability of cells to migrate through a Matrigel basement membrane matrix was measured using the Cell Invasion Assay Kit (Chemicon International, Temecula CA) with 8 µm pore size polycarbonate membrane. After rehydration of the gels for 2 hours, 1×10^5^ cells in serum-free DMEM were applied to the upper chamber while the lower chamber contained serum-free DMEM or serum-free DMEM supplemented with CCL18 in various concentrations or TGFbeta as a control. After incubation at 37°C for 24 hours, all cells of the upper chamber side were removed with a cotton swab and the membrane was removed and the invasive cells were stained using cristal violet. Analysis was performed by counting 9 high-power fields per filter at 200-fold magnification using a Olympus microscope (Olympus, Tokio, J.P.N.).

### Chemoresistance Assay

To measure the influence of CCL18 on chemoresistance of the tumor cells, A549 cells were cultured in a 6-well plate and grown to 80% confluence. Cells were cultured for 24 and 72 h with or without CCL18 at concentrations as indicated. Subsequently, a dilution series of Cisplatin ranging from 0.4 to 25 µg/ml was added for additional 72 hours. At the end of the assay period, living cells were measured using MTT [3-(4,5-dimethylthiazol-2-yl)-2,5-diphenyl-tetrazolium bromide] assay. Medium was changed and MTT solution (5 mg/ml in phosphate buffered saline/well) was added for 4 h and the generated formazan was solubilized in isopropanol and measured spectrophotometrically at 563 nm. The optic density retrieved for non-treated cells was set 100% and used to calculate the percentages of viable cells in the treated cultures.

### BRDU -Proliferation assay

DNA synthesis was measured using a bromodeoxyuridine (BrdU) Cell Proliferation Kit (Calbiochem, Darmstadt, FRG). BrdU labeling solution was added to the cells in combination with the different treatments and incubated for 24 h. After removal of the culture medium, the cells were fixed, permeabilized and the DNA was denatured via microwave. Anti-BrdU antibody was added for one hour prior to the addition of mouse IgG–peroxidase conjugate for 20 minutes. The signal was developed with tetramethylbenzidine solution in darkness. Absorbance was measured using a spectrophotometric plate reader at 450 nm with a reference wavelength at 595 nm.

### Statistical analysis

Statistical analysis was conducted by using StatView (SAS Institute, Cary, NC). Values are shown as mean±SD of at least of a minimum of three independently conducted experiments. Dose-dependencies were calculated by Friedman Test followed by paired comparisons using the Wilcoxon Test.

## Results

### Change of Cell Morphology

**Figure 1:**
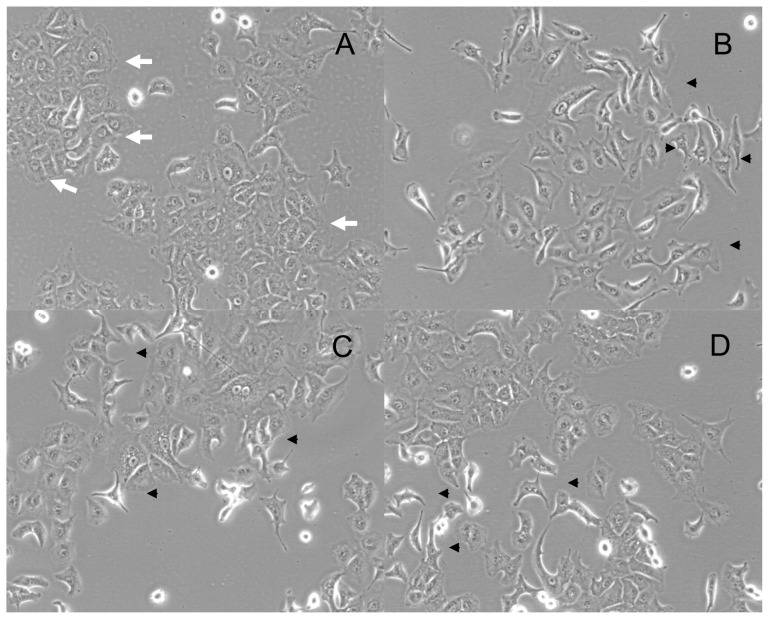
Morphological changes in cells treated with CCL18 and TGFbeta: untreated A549 cells show typical epithelial morphology with close cell-cell-contacts, which are marked by white arrows (A). After treatment with 2 ng/ml -β cells changed the phenotype to a more mesenchymal morphology with prolonged cell extrusions and loosened cell-cell-contacts marked by arrowheads. (B) CCL18 also changes the cell morphology in a dose dependent manner (C [1 ng/ml] and D [10 ng/ml]) to fibroblast-like mesenchymal phenotype and loosened cell-cell-contacts marked by arrowheads.

Initially, A549 disclosed a typical cuboid epithelial morphology with tight cell-cell contacts (Fig. 1A). Treatment of the cells with CCL18 for 24 hours induced marked changes in cell morphology (Fig. 1C and D). Cells changed their morphology from cuboid or triangle-shape to spindle-shape; developed prolonged cell extrusions and cell-cell contact were less intense. These changes could be observed over a dose range from 1 to 100 ng/ml (Fig. 1C). As expected, stimulation with 2 ng/ml TGFbeta for 24 hours induced also a spindle-shape, fibroblast-like phenotype (Fig. 1B). Thus, CCL18 and TGFbeta induce morphological changes compatible with EMT.

### Expression of EMT-related Genes

**Figure 2:**
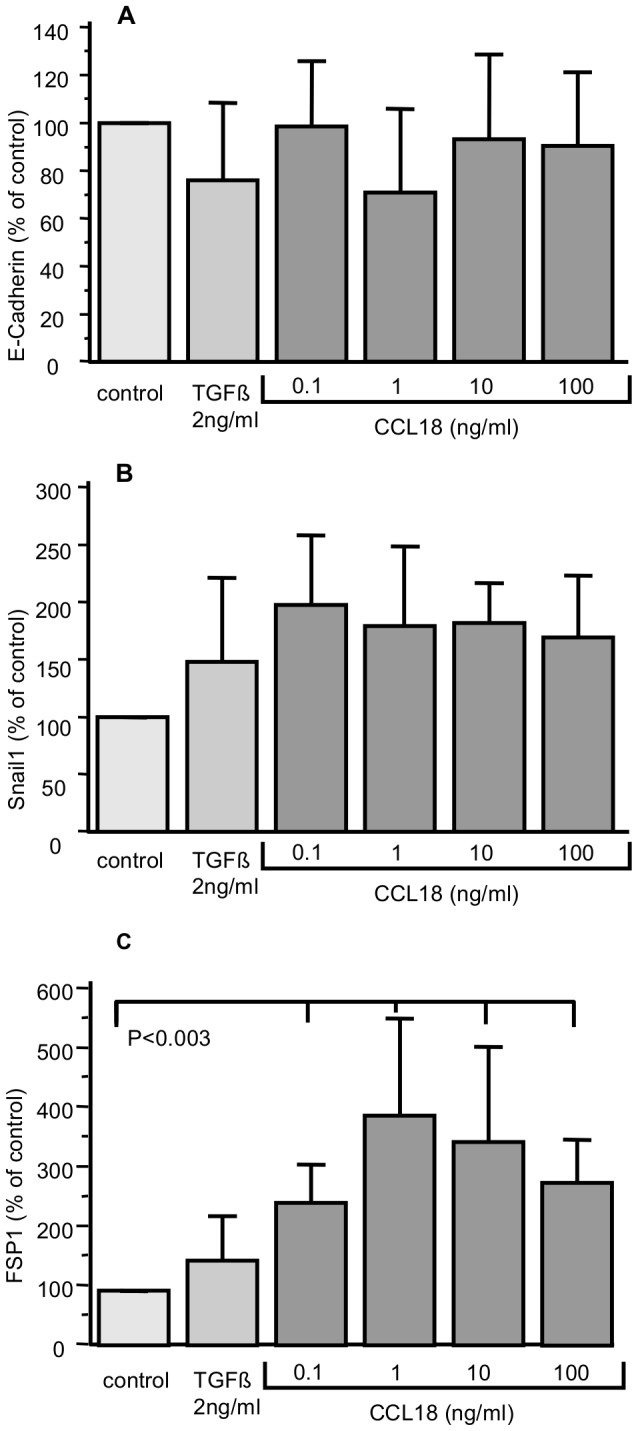
mRNA levels of (A) E-cadherin, (B) SNAIL1, (C) FSP-1 after stimulation with -β or various CCL18 concentrations for 72 h relative to the non-stimulated control. Each bar represents means ±SD of four independent experiments. GAPDH was used as housekeeping gene to normalize for differences in the amount of total RNA.

Using RT-PCR, we could not detect any clear-cut effect of CCL18 on E-cadherin expression in any of the concentrations used after a stimulation time of 72 hours (Fig. 2A). In contrast, stimulation of A549 with CCL18 for 72 hours increases SNAIL1 (Fig. 2B) and FSP-1 expression (Fig. 2C). The increase in FSP-1 expression was statistically significant (p<0.03). Interestingly, the effect was maximal at 1 ng/ml CCL18 and decreased slightly with increasing concentrations (Fig. 2C.). Compared to control CCL18 stimulation almost doubled mean SNAIL1 expression. However, this increase did not reach significance and no clear dose-relationship could be detected (Fig. 2B). Interestingly, although TGFbeta induced an increase in SNAIL1 and FSP1 expression, the effects were lower than observed with the comparable CCL18 concentration (1 ng/ml). Again, no effect on E-cadherin expression could be found.

### Changes in the Expression of EMT-related Proteines

**Figure 3:**
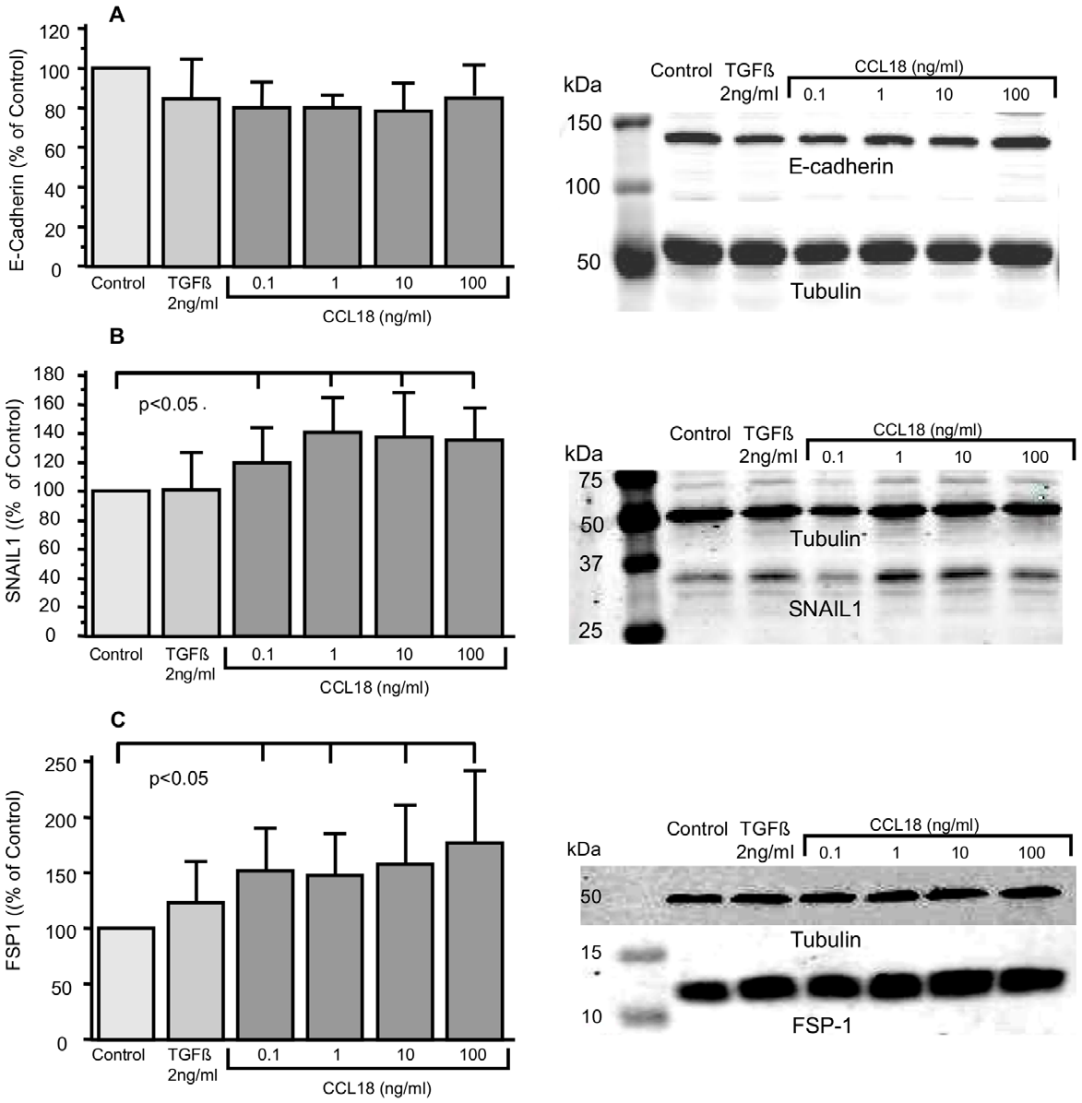
Effects of CCL18 on the expression of EMT-related proteins (SNAIL1, E-cadherin and FSP-1) after 72 hours of treatment. All results were normalized to the untreated control. Bars summarize the results of three independent western blot analyses, in the right panel one representative blot is depicted.

To confirm our PCR findings, we analyzed protein expression by Western Blot. In parallel with the PCR results, E-cadherin expression disclosed a slight but non-significant decrease when cells were stimulated with CCL18 for 72 hours (Fig. 3A). In contrast, we found significantly increased SNAIL1 (p<0.005; Fig. 3B) and FSP-1 expressions (p<0.05; Fig. 3C) by forty and fifty percent, respectively. TGFbeta (2 ng/ml) induced either no (snail1) or only marginal and insignificant changes (E-cadherin, FSP1, Fig. 3).

### CCL18 related Chemotaxis and Invasion

**Figure 4:**
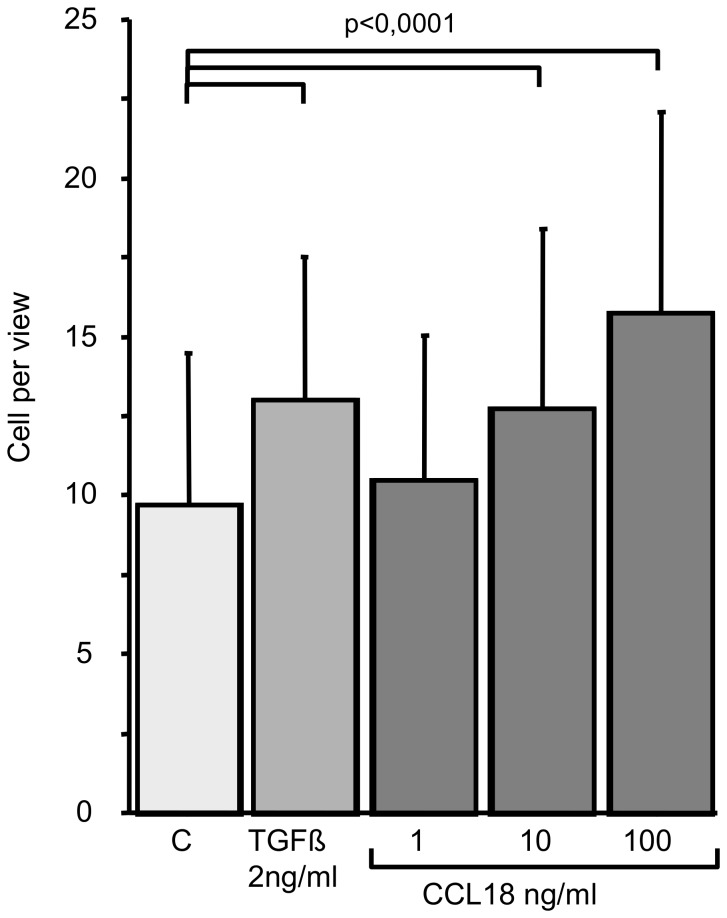
CCL18 induces chemotaxis in A549 lung cancer cells. Significances were calculated using paired comparison (Wilcoxon Signed Rank Test), control: untreated cells (n = 4).

**Figure 5:**
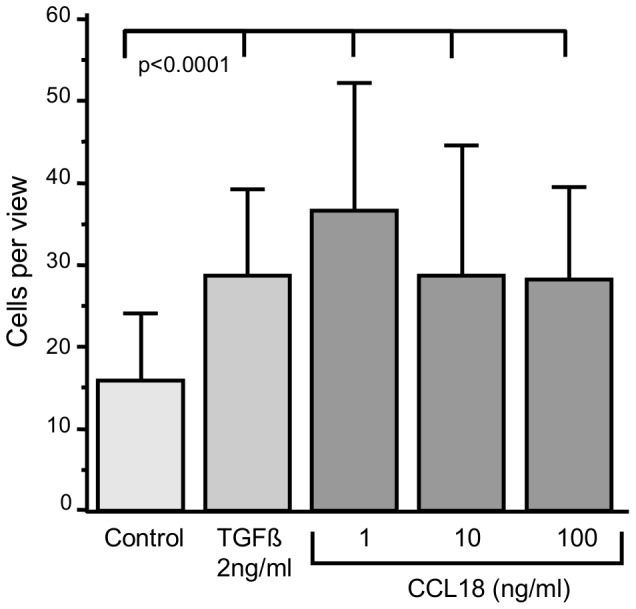
CCL 18 induces invasion of A549 lung cancer cells into a extracellular matrix derived from the Engelbreth Holm-Swarm (EHS) mouse tumor. Significances were calculated using paired comparison (Wilcoxon Signed Rank Test), control: untreated cells (n = 3).

Boyden chamber experiments revealed that CCL18 induces chemotaxis of A549 cells in a dose-dependent manner. We observed a dose dependent and significant (p<0.0001) increase in chemotaxis induced by CCL18, which reached its maximum (50% over control) at a dose of 100 ng/ml CCL18 (Fig. 4). TGFbeta (2 ng/ml) enhances the chemotaxis by 40% which was also statistically significant (p<0.0001). The gain of the capability for invasion into a tissue or matrix is a further and important characteristic of EMT requiring proteolytic activity additional to chemotaxis. Therefore, we analyzed the effect of CCL18 on the ability of invasion. We found that CCL18 (1 ng/ml) increases invasion more than twofold compared with control (p<0.0001; Fig. 5). TGFbeta (2 ng/ml) increases invasive in the same range but less pronounced (p<0.0001).

### Proliferation

**Figure 6:**
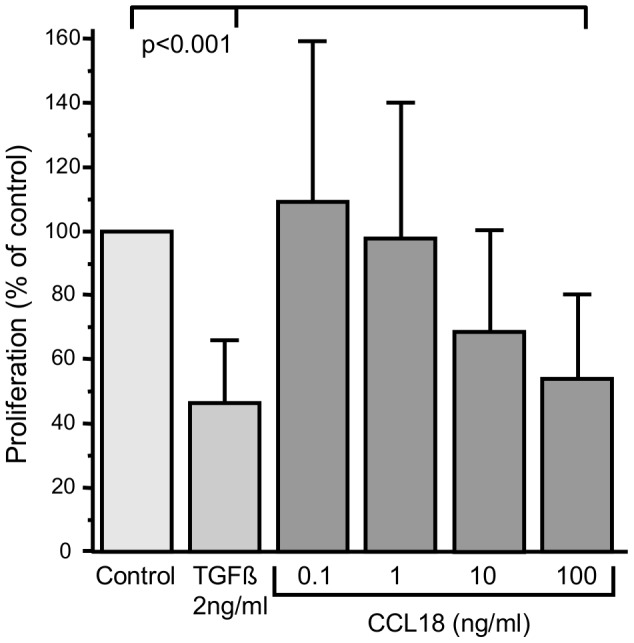
CCL18 suppreses proliferation of A549 cells. (n = 7 in each group).

To determine the effect of CCL18 on cell proliferation, A549 cells were treated for 24 hours with either 2 ng/ml of TGFbeta or 0.1 ng/ml, 1 ng/ml, 10 ng/ml, or 100 ng/ml of CCL18, respectively, and subsequently a BrdU assay was performed. Incubation with CCL18 decreased the proliferation rate in a dose dependent manner reaching a significant reduction of 50% compared with controls at 1000 ng/ml (p<0.001; Fig. 6). TGFbeta (2 ng/ml) reduced the proliferation rate by 55% compared with controls (p<0.001).

### Chemoresistance

**Figure 7:**
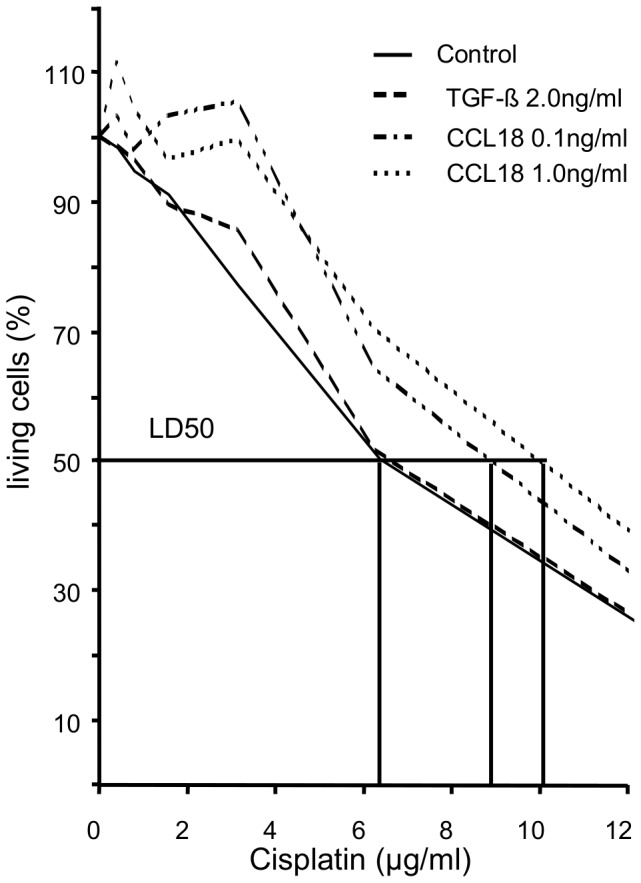
Influence of CLL18 on chemoresistance. Cells were cultured in presence or absence of -TGFbeta or CCL18 in the concentrations indicated for 72 h. After the culture period, medium was changed and the cells were incubated with cisplatin for additional 72 hours at concentrations ranging from 0.4 to 25 µg/ml. Cell survival was measured by MTT assay. The horizontal line indicates the LD_50_, vertical lines indicate the LD_50_ for the respective pre-incubation condition (n = 4).

We further investigated if CCL18 modulates the sensitivity of A549 cells to Cisplatin treatment. Therefore, we incubated the cells with CCL18 and subsequently treated the cells with serial dilutions of Cisplatin. After CCL18 stimulation for 72 hours, no toxic effects of CCL18 or TGFbeta could be detected (data not shown). LD_50_ of untreated A549 cells was 6 µg/ml Cisplatin. After CCL18 stimulation (0.1 ng/ml) of the cells for 72 hours, the LD_50_ increased to 9 µg/ml Cisplatin. Likewise, the LD50 of stimulated cells rose to 10 µg/ml when the cells were pretreated with 1 ng/ml CCL18 (Fig. 7). Higher concentrations of CCL18 were tested but no additional effect was seen (data not shown).

## Discussion

Lung cancer is one of the malignancies with the highest incidence in the Western world and despite all advances in diagnosis and therapy one of the leading causes of cancer related death [23]. Nearly 85% of these patients suffer from non-small cell lung cancer (NSCLC), which is subdivided into squamous carcinoma, adenocarcinoma, large cell carcinomas and other rare subtypes [24]. Adenocarinoma represents the highest proportions of NSCLC and since the 1970s its proportion is increasing [25]. At time of diagnosis the majority of patients with NSCLC suffer already from advanced or metastatic disease, which is associated with poor prognosis [26]. The mechanism of metastasis in lung cancer is not fully understood, but epithelial to mesenchymal transition (EMT) seems to be one key event in the process of metastasis. Some authors already demonstrated that various mediators released in the microenvironment of solid tumors are able to induce EMT and therefore promote disease progression [14]. CCL18 is a chemokine produced and released by tumor-associated macrophages (TAM) in the microenvironment of cancer cells and pulmonary fibrosis [16], [17]. We could demonstrate that CCL18 is highly elevated in patients with NSCLC and correlates with T-stage and mean survival time in the adenocarcinoma subgroup [21]. This leads to the question if CCL18 is directly involved in pathogenic processes in cancer diseases. Since CCL18 was found to induce collagen in lung fibroblasts and to act in a similar way as TGFbeta [17], [27], [28], we hypothesized that CCL18 may promote the process of metastasis via EMT. A549 cells are a well characterized human cell line isolated from an adenocarcinoma of the lung, which has already been used in several studies investigating the induction of EMT by TGF-β [29], [30]. In agreement with others, we demonstrate that A549 cells treated with even low dose of TGF-β underwent morphological changes from cuboid epithelial cells to spindle shaped fibroblast-like cells and acquired a mesenchymal phenotype after 24 hours of treatment. Likewise, we were also able to demonstrate that CCL18 induces the same morphological changes like TGF-β. This is further supported by analysing the expression pattern of epithelial and mesenchymal genes on mRNA and protein level. The up-regulation of mesenchymal genes like FSP-1 or SNAIL1 is a hallmark in the process of EMT. The observed changes of the characteristic expression pattern were quite similar in TGF-β and in CCL18 stimulations. In this context, it is notable that Miyazaki et al. published in 2006, that patients with pulmonary adenocarcinoma demonstrating a low E-cadherin expression in combination with a high FSP-1 expression have to face a significant poorer prognosis due to hematogenous metastasis [9]. We also observed that SNAIL1, a transcription factor involved in the early events of EMT, was up-regulated by CCL18 treatment in the same way as by TGFbeta. In the process of metastasis, cancer cells acquire new capabilities like invasion, enhanced migration and down-regulation of proliferation. We, therefore, studied also the influence of CCL18 on these cell functions and observed an enhanced migratory and invasive potential after 24 hours of CCL18 treatment. Moreover, the proliferation rate of A549 decreased after 24 hours of CCL18 treatment, which might be due to the initiation of differentiating processes. In total, these broad changes in cell functions mentioned above demonstrate a strong influence of CCL18 in the process of metastasis. Thus, based on our data presented here, on our previously published data that increased serum CCL18 correlates with decreased survival time in adenocarcinoma of the lung [22] and on the observation by Chen et al. demonstrating that CCL18 promotes metastasis in breast cancer [5] we hypothesized that CCL18 may be one of the key factors driving EMT in the tumor environment.

Keeping in mind that the majority of patients require chemotherapy either as adjuvant or neoadjuvant part of a multimodal approach or as a single therapy, chemoresistance is still one of the main barriers in sufficient treatment of NSCLC and the reason of tumor progress, relapse and tumor death. Chemotherapy of NSCLC still relies on platinum derived drugs although it is known that several mechanisms exist how lung cancer cells escape cisplatin cytotoxicity and CCL18 might be a pivotal mediator in these processes. These mechanisms seem to be linked to EMT because transcription factors regulating EMT also regulate transporter proteins related to an increased resistance to platinum agents [31], [32]. Therefore, we investigated the role of CCL18 in chemoresistance. We observed indeed an increased chemoresistance induced by CCL18. Interestingly, this increase in chemoresistance peaked in the same range as markers of EMT (e.g. FSP1). This provides evidence for a role of CCL18 in the induction of chemoresistance. Because CCL18 is a chemokine, which might also be involved in the homing of cancer cells, we also investigated the chemotacic properties of CCL18 on A549 cells. We observed that CCL18 exerts a dose dependent increase in chemotaxis of lung cancer cells indicating, that CCL18 attracts lung cancer cells to sites of CCL18 release. As the lung is the organ with the highest level of CCL18 production, this effect might influence the homing of CCL18 responsive tumor cells to the lung.

Interestingly, in most of the experiments we could not observe a clear-cut dose-dependent effect of CCL18. In contrast, increasing the concentrations of CCL18 often results in decreasing effect of CCL18. This is compatible with the expression of different agonistic and antagonistic receptors of CCL18. Recently, Catusse et al. published that CCL18, triggering via GPR30, acts agonistic and diminishes CXCR4-mediated effects of CXCL12 [15]. Chen et al. demonstrated that PITPNM3, a membrane-associated phosphatidylinositol transfer domain-containing protein, might act as a CCL18 receptor [5]. We identified the already known chemokine receptor CCR6 as a CCL18 receptor with potency to initiate the described activities in fibroblasts [33]. This indicates that CCL18 might use several receptors and the balance of the receptor expression might regulate the agonistic and antagonistic effects of CCL18.

In conclusion, our data demonstrate that CCL18 induces EMT in A549 cells, enhances their metastatic potential and supports chemotherapy resistance. Thus, CCL18 is not only a mediator released by tumor-associated macrophages and potentially reflecting the tumor size but it also influences neoplastic processes of adenocarcinoma. The data presented here demonstrate that CCL18 is able to trigger events involved in tumor metastasis such as change from primary epithelial tumor cells into migratory mesenchymal cells, chemotaxis, and invasion. In addition, CCL18 also protects tumor cells from chemotherapy by increasing chemoresistance. Thus, we conclude that CCL18 will be a future target in the treatment of lung cancer.
